# Recent advances in model systems for interrogating diseases of brain aging and associated dementia: Toward human-relevant endophenotypes

**DOI:** 10.1016/j.conb.2025.103138

**Published:** 2025-11-13

**Authors:** Stacey J. Sukoff Rizzo

**Affiliations:** 1 Aging Institute, University of Pittsburgh School of Medicine, Pittsburgh, PA, USA; 2 Department of Neurobiology, University of Pittsburgh School of Medicine, Pittsburgh, PA, USA

## Abstract

Neurodegenerative diseases are characterized by key pathological hallmarks, progressive loss of neuronal structure and function, and synaptic loss. Often, mild behavioral changes including subjective cognitive decline and neuropsychiatric symptoms precede the diagnosis and may be a harbinger of disease inception and progression. Despite the success of new treatments that attenuate pathological burden, the ability to translate clinical benefit for cognitive impairment and dementia-related behavioral syndromes remains challenging. While model systems are essential, the appropriate model must be carefully chosen for the specific research question, with complementary model systems necessary to capture multiple aspects of disease. This review will cover the emergence of model systems that provide more translationally relevant trajectories of the progression of pathological changes throughout brain aging, and the advancement of model systems that are able to better capture the spectrum of behavioral and cognitive changes that signal the early prodromal period prior to diagnosis.

## Introduction

Dementia affects over 57 million people worldwide and is a major cause of disability and dependency among older adults, and with no clear prevention or treatment available to date [1,2]. Alzheimer’s disease (AD) is the most common cause, but dementia is also prominent in all neurodegenerative disorders including Parkinson’s disease (PD) and Amyotrophic Lateral Sclerosis (ALS), as well as other forms of dementia such as frontotemporal dementia (FTD), Lewy body dementia (LBD), and vascular dementia (VaD) [3,4]. While there has been substantial progress on disease modifying therapies, treatment of the behavioral and cognitive symptoms remains challenging. There are currently no non-in vivo alternative models that capture complex human behaviors that are altered in diseases of brain aging. Regardless of emerging interests in artificial intelligence to replace in vivo models, these are unlikely to identify a cure for the behavioral symptoms and cognitive impairment that contribute to dementia until an intervention that stops and prevents these changes has been achieved to allow for better in silico predictions. This review will cover two major advancements in the field: the emergence of model systems that provide more translationally relevant trajectories of the progression of pathological changes throughout brain aging, and the advancement of model systems that are able to better capture the spectrum of behavioral and cognitive changes that signal the early prodromal period prior to diagnosis.

## Symptom clusters in dementia and the utility of model systems in recapitulating disease and behavioral endophenotypes

**Figure 1:**
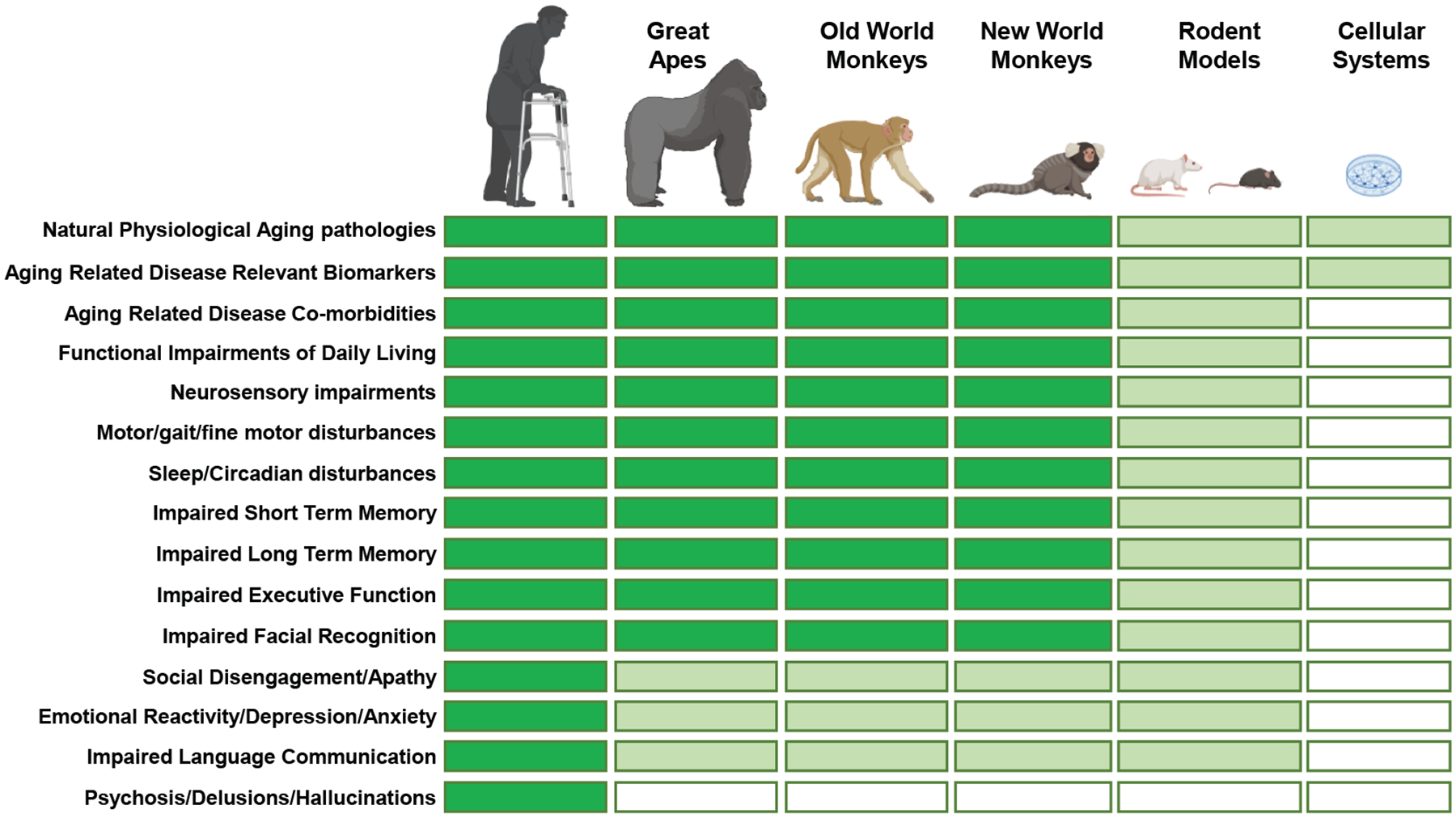
Recapitulation of dementia endophenotypes in model systems across the evolutionary spectrum. Shading of boxes under each representative model system reflects the limits of construct validity of each measure relative to human. For example, long term memory cannot be captured in cellular systems or limited to face validity in short lived species such as rodents: Unshaded = cannot be adequately assessed; light green shading = partial recapitulation relative to human endophenotype/limited to face validity; Dark green shading = recapitulation of human endophenotype. Illustrations created using BioRender.com.

While dementia is the general term used to characterize the syndrome of behavioral and cognitive changes that are associated with neurodegenerative disorders, each presents with overlapping and distinct clinical profiles [3,4]. These syndromes manifest as symptom clusters, or *endophenotypes*, that include cognitive impairment and functional deterioration as well as the non-cognitive behavioral and psychological symptoms of dementia often referred to as BPSD [5,6]. Related to the, cognitive endophenotypes of dementia, these include: memory impairment (episodic, semantic), executive dysfunction (planning, behavioral flexibility), visuospatial dysfunction (facial recognition, depth perception, spatial navigation), and language impairments (naming, fluency). BPSD symptom clusters include: apathy/anhedonia (social disengagement), agitation and aggression, depression and anxiety, and psychosis (delusions, hallucinations). While functional endophenotypes include motor deficits (gait, motor disturbances), and decline in activities of daily living including self-care. These symptom clusters are not only relevant for diagnosis of the different types of dementia associated with disease of aging, but also as markers for disease progression and for assessing efficacy of clinical interventions [6–9]. To develop effective interventions for dementia, preclinical models must not only recapitulate disease pathology, which is the biological targets of interventions, but must also faithfully reproduce these symptom clusters. Notwithstanding, it is unlikely that any one model will be sufficient to recapitulate all aspects of the spectrum of endophenotypes of any given disorder. Moreover, several behavioral endophenotypes are uniquely human (e.g., delusions, semantic language) and are unlikely able to be modeled in any other species but humans (Figure 1). Models should be selected to address the question they are best suited for; and researchers need to be cautious about forcing overinterpretations of model systems to capture multiple dimensions of such complex components of dementia-related disorders that manifest differently across individuals.

## Dementia in a dish: the benefits and limitations of cellular model systems

Cellular model systems including induced pluripotent stem cells (iPSCs) and organoids have revolutionized research in neurodegenerative disorders. Dementia patient-derived iPSCs can be differentiated into disease-relevant cell types, such as neurons, astrocytes, and microglia to evaluate disease-specific phenotypes, including protein aggregation and cellular dysfunction; as well as used for drug screening to identify novel targets specific to these cell types and for high-throughput screening platforms [10–12]. Relatedly, CRISPR/Cas9 tools can be used to generate isogenic iPSC lines to introduce disease mutations as well as tools for testing therapeutic hypotheses in modulating these disease related signatures [13,14]. Aside from iPSCs, the age and molecular disease signature of the cells can also be preserved by direct conversion to neurons and astrocytes even from skin biopsies derived first into fibroblast lines or from peripheral blood mononuclear cells (PBMCs), which have been shown to be an excellent surrogate for brain at the transcriptome and proteome levels [13,15,16]. In addition to single cell platforms, there have been recent advancements in organoids as three-dimensional co-culture systems that not only include neurons, astrocytes, and microglia, but also capture critical elements of brain vascularization including brain microvascular endothelial cells, pericytes, and oligodendrocyte precursor cells [17,18]. While these systems can also be used to identify targets of disease, capture biomarkers from secreted proteins in media, and have functionality to measure synaptic and neuronal integrity using electrophysiological approaches, cell systems cannot be characterized as having behavioral features of dementia for which in vivo models remain necessary. Thus, complementary models must be used to test hypotheses generated from these cellular systems, especially to evaluate prodromal dementia and early behavioral changes that manifest prior to disease pathology.

## Rodent models: challenges and limitations for translating dementia endophenotypes

Rodent models have been central to modeling the genetic and environmental risk factors for dementia. Genetic engineering approaches in rodents have been necessary to enable the study of the aggregation of pathological proteins such as tau, amyloid, alpha-synuclein, and other proteins that do not aggregate naturally in rodents. More specifically, genetic engineering tools have allowed the ability to study protein misfolding and aggregation including forming inclusions that are characteristic of AD, PD, and other neurodegenerative diseases. Notably, transgenic overexpression of human amyloid precursor protein (APP), presenilin mutations (PSEN), and tauopathies which have dominated the field, are also associated with overexpression artifacts and many of these models do not exhibit neurodegeneration or progression of key pathologies resembling the same progression documented in the human disorders [19–21]. Relatedly, the transgenic overexpression often occurs early in the lifespan of the mice, during adolescence, which does not align with the analogous human age of onset for neurodegenerative disorders. Irrefutably, while these models have been successful in informing target engagement for therapeutic interventions including allometric scaling of dosing for translational pharmacokinetic and pharmacodynamic studies, they fall short in their translational application for improvements in cognitive function and dementia endophenotypes. Nearly all transgenic rodent models become hyperactive with progression of pathology, another likely artifact of transgene over-expression, and this hyperactivity is an important confound in the interpretation of behavioral and cognitive tasks which largely rely on activity measures [22–24]. Numerous publications report deficits in learning and memory; many of these publications evaluate rodent behavior in the water maze, novel object recognition, or fear conditioning assays, which are incomplete analogs for complex human cognitive functions and highly influenced by hyperactivity which confounds the ability of the mice to perform these tasks [22–24]. Recent data from knock-in mouse models are reported to have minimized the hyperactivity artifacts [25–27], which coupled with more translationally relevant tasks such as touchscreen cognitive testing batteries, are allowing improved paradigms for investigating cognitive changes in rodent models [25–27]. Whether rodents have analogous executive function and long-term episodic memories to humans remains a topic of considerable debate given their lack of key anatomical cytoarchitecture in the human prefrontal cortex [28,29]. Critically, therapeutic interventions that have demonstrated improvements in these cognitive domains including variations in water maze, novel object, and fear conditioning tests, as well as other rodent cognitive behavioral tasks, have failed to show improved clinical benefit for cognitive function and dementia in human patients, thus limiting the predictive validity and translational validity of these rodent behavioral assays [22]. Indeed, rodent models have been successful with predictive validity for therapeutic interventions for treating anxiety and depression in the general context of neuropsychiatric disorders, though the assays and measurements used such as immobility in the forced swim test or thigmotaxis behavior in an open field, for example, have limited translational validity to human affective states [22,24,30,31]. In addition, the ability to detect subtle behavioral changes and mild cognitive impairment in rodents prior to pathology has been challenged by the limited aging timeframe, especially in overexpression models, where the onset of pathology is often present early during adolescence in the mice. Interestingly, the newest generation of genetically engineered models that avoid transgenes (e.g, CRISPR/Cas9) with a focus on late onset sporadic AD (LOAD) genetic risk coupled with environmental exposures, indeed have much milder phenotypes and are valuable for studying the early prodromal stages of disease prior to significant amyloid or tau aggregation [32,33]. Noting that significant amyloid and tau deposition, which are so desirable by many researchers is also a recapitulation of the latest stages of the human disease. Thus, a paradigm shift is needed by both researchers and reviewers to better appreciate the ability to identify subtle changes that harbinger the inception and progression of disease. More specifically, the ability to demonstrate subtle but incremental changes with aging may have improved translational relevance for modeling neurodegenerative disease than statistically significant overexpression of amyloid and tau during the adolescent and early adult stages of the rodent lifespan. Validation of subtle and incremental changes has already been established in the clinic to stage the earliest signs of disease, where rating scales such as the Clinical Dementia Rating Sum of Boxes (CDR-SB) have been developed, specifically to be more sensitive to incremental changes in cognitive and functional abilities, particularly in the early stages of AD including mild cognitive impairment [34]. While cumulative rating scales have not yet been established for non-human models, we propose that mild non-statistically significant changes that present incrementally with aging, across correlating outcomes (e.g., behavior, pathology, gene expression, protein expression, synaptic alterations), and that can be demonstrated as reproducible across laboratories should be highly valued over single data points with p-value <0.05 reported in only a single lab [35]. Therefore, while rodent models continue to remain valuable to address specific research questions, animal models with longer lifespans that can better recapitulate the spectrum of sophisticated human behaviors and cognitive domains that can be tested longitudinally to capture the long prodromal period prior to the inception and progression of disease provide complementary models to those of the types of studies that can be readily conducted and are well validated in rodent and cellular model systems.

## Non-human primates as essential model systems for studying the spectrum of behavioral and cognitive symptoms of dementia

Our understanding of the causes of behavioral and cognitive changes that precede the diagnosis of dementia have been limited by two major barriers: 1) that individuals do not visit the clinic until there is already a medical issue limiting the ability to study the inception and progression of these symptoms, and 2) that we have not been using the most relevant model systems for addressing questions related to the behavioral and cognitive features of dementia. More specifically related to cognitive function and endophenotypes associated with the behavioral symptoms of dementia, primate models (both human and non-human) are essential (Figure 1). Advancing through the phylogenetic scale, from New World monkeys such as marmosets and capuchins, through Old World monkeys such as macaques and baboons, and towards great apes, non-human primates (NHPs) share evolutionary conservation of genes, proteins, behaviors, and brain anatomy and function with humans that is greater than that of non-primate models [36–41]. NHPs present with a repertoire of complex and sophisticated behaviors that mirror those of humans and change with aging including affective, reactive, and social and affiliative behaviors [40–45]. Many of these behaviors are primate specific and capture the spectrum of behaviors associated with BPSD in humans (Figure 1). Beyond their behavioral similarities related to dementia both new-world and old-world NHP species show aging related hippocampal atrophy, impaired cognitive function, and decreased prefrontal function, paralleling features of prodromal dementia [46–50]. Related to cognitive function, the near identical touchscreen-based cognitive assessments used to detect mild cognitive impairment and cognitive decline in patients have been validated across NHP species. More specifically, comprehensive cognitive testing batteries have been established in NHPs that capture the full spectrum of cognitive domains including short and long term memory, reversal learning, attention, recognition memory, and executive function; which have been a limited capability in rodent models [46–51]. Further, aging NHPs show natural and sporadic presentations of amyloid beta (Aβ) deposits, neurofibrillary tau tangles and vascular lesions; with recent emerging data also now revealing the natural presence of mixed co-pathologies with aging including alpha-synuclein and TDP-43 that are characteristic features of the brain pathologies of dementias [52–56]. Importantly, because of their long lifespan, the timeline for close investigation of the behavioral changes that precede aging-related cognitive decline as well as biomarkers of disease is possible in NHPs. Moreover, with careful observations from birth throughout the lifespan, there is a unique opportunity to study inception and progression of disease under controlled environmental conditions with well documented medical and nutritional records which cannot be conducted in humans.

## Great ape species as model systems to study the susceptibility and resilience to AD and related dementias in humans

While non-human primate studies of AD have traditionally been conducted in laboratory environments, there is a benefit to studying our closest evolutionary relatives, the great apes, in their natural free-ranging environments. More specifically, the ability to study great ape species including chimpanzees, bonobos, gorillas, and orangutans in their natural habitats, and in comparison to these same species in their captive and subsidized environments (e.g., zoos, conservation centers), is poised to reveal mechanisms that drive genetics x aging x environment susceptibility and resilience to dementia. Great apes like other primate species have similar brain pathologies to humans though access to tissue has been limited which has made it challenging to fully investigate the comparability of frank neurodegeneration with humans. Comparative evolutionary genomics studies have provided invaluable insight into the biological processes that differentiate humans from other primate species with respect to aging and neurodegeneration [57,58]. Behavioral observations in both free-ranging and zoo populations of great apes reveal comparative aging related changes to humans including motor slowing, declines in executive function tasks, and distinct social changes [59–62]. Other human behaviors that are also distinctly observed in great ape and other NHP species include analogs of empathy and facial recognition [63–65]. Additionally, wild great ape species often face environmental stressors analogous to humans including early life adversity and disabilities that allow for the investigation of behavioral consequences and susceptibility to diseases of aging in later life [56–60]. Although more challenging in their natural environments, great ape behaviors have been successfully studied at the individual level non-invasively by field biologists tracking individuals through non-invasive analysis of genomic DNA and biomarkers from feces [66]. Taken together these findings emphasize the value of investigating behaviors and comparative evolutionary genomics in great ape and NHP species to understand the mechanisms that drive resilience to dementia, thus enabling interventions targeting molecular targets that may provide protection against neurodegeneration and dementia in humans.

## Concluding remarks

Complementary model systems have provided invaluable contributions to advancing our understanding of the broad spectrum of molecular, biochemical, pathological, and behavioral endophenotypes of dementia. Moving beyond traditional transgenic mouse models is crucial to enhance translational relevance. Combining iPSCs, organoids, CRISPR, and more translationally relevant animal models that recapitulate primate-specific behaviors offers unprecedented opportunities to unravel disease mechanisms, particularly those underpinning dementia and neuropsychiatric symptoms. Non-human primates with their behavioral, cognitive and neuroanatomical proximity to humans, offer promising and advantageous evolutionary conservation for modeling symptom clusters and advancing therapeutic translation in dementia beyond the limitations of cellular and rodent models. A paradigm shift toward endophenotype-driven modeling and cross-species validation is essential for progress in dementia research.

## Data Availability

No data was used for the research described in the article.
